# Tumor suppressor HIC1 is synergistically compromised by cancer-associated fibroblasts and tumor cells through the IL-6/pSTAT3 axis in breast cancer

**DOI:** 10.1186/s12885-019-6333-6

**Published:** 2019-12-03

**Authors:** Xueqing Sun, Qing Qu, Yimin Lao, Mi Zhang, Xiaoling Yin, Huiqin Zhu, Ying Wang, Jie Yang, Jing Yi, Mingang Hao

**Affiliations:** 1 Department of Biochemistry and Molecular Cell Biology, Shanghai key Laboratory of Tumor Microenvironment and Inflammation, Shanghai Jiao Tong University School of Medicine, Shanghai, 200025 China; 2 Department of Oncology, Shanghai Jiao Tong University School of Medicine, Ruijin Hospital, Shanghai, 200025 China; 3 Institution of Life Science, Chongqing Medical University, Chongqing, 400016 China; 4 Department of Otolaryngology Head and Neck Surgery, Shanghai Ninth People’s Hospital, Shanghai Jiao Tong University School of Medicine, Shanghai, 200011 China; 5 Department of Cancer Biology, University of Cincinnati College of Medicine, Cincinnati, OH 45267 USA

**Keywords:** Breast cancer, CAF, IL-6, STAT3, HIC1

## Abstract

**Background:**

Interleukin-6 (IL-6) is commonly highly secreted in the breast cancer (BrCA) microenvironment and implicated in disease development. In this study, we aimed to determine the role of the IL-6/pSTAT3/HIC1 axis in the breast cancer microenvironment, including in cancer-associated fibroblasts (CAFs) and breast cancer cells.

**Methods:**

Stromal fibroblasts from the breast cancer tissue were isolated, and the supernatants of the fibroblasts were analyzed. Recombinant human IL-6 (rhIL*-*6) was applied to simulate the effect of CAF-derived IL-6 to study the mechanism of HIC1 (tumor suppressor hypermethylated in cancer 1) downregulation. IL-6 was knocked down in the high IL-6-expressing BrCA cell line MDA-MB-231, which enabled the investigation of the IL-6/pSTAT3/HIC1 axis in the autocrine pathway.

**Results:**

Increased IL-6 was found in the supernatant of isolated CAFs, which suppressed HIC1 expression in cancer cells and promoted BrCA cell proliferation. After stimulating the BrCA cell line SK-BR-3 (where IL-6R is highly expressed) with rhIL-6, signal transducers and activators of transcription 3 (STAT3) was found to be phosphorylated and HIC1 decreased, and a STAT3 inhibitor completely rescued HIC1 expression. Moreover, HIC1 was restored upon knocking down IL-6 expression in MDA-MB-231 cells, accompanied by a decrease in STAT3 activity.

**Conclusions:**

These findings indicate that IL-6 downregulates the tumor suppressor HIC1 and promotes BrCA development in the tumor microenvironment through paracrine or autocrine signaling.

## Background

Breast cancer (BrCA) is one of the most common malignant tumors in women and the leading global cause of tumor prevalence and death in women [1]. According to statistics from the National Cancer Registry of China in 2015 [2], there were approximately 269,000 cases of breast cancer and approximately 70,000 deaths, accounting for 15 and 7% of female morbidity and mortality, respectively. BrCA can be intrinsically clustered into five subtypes including Luminal A (L-A), Luminal B (L-B), Her2-overexpressing (Her2-oe), triple-negative (TNBC) and normal-like breast cancer based on the gene expression profile [3], while the first four subtypes are commonly used in studies [4].

The tumor microenvironment refers to a locally stable environment in which tumor cells, macrophages, fibroblasts, vascular endothelial cells, immune cells, and extracellular matrix exist together and benefit tumor development and metastasis [5]. Cancer-associated fibroblasts (CAFs) are the most abundant cell types in the tumor microenvironment; they secrete various cytokines, such as CXCL12, IL-1, IL-8, IL-10, IL-6, TNF-α and MCP-l, through the paracrine pathway to act on tumor cells and promote tumorigenesis and the development of the tumor [6–10].

In this work, we found that CAFs derived from the four different pathological types of BrCA tissues have common features regarding the high secretion of IL-6, IL-8 and GRO (CXCL1, 2, 3) (see results). IL-6 is one of the most versatile cytokines involved in the regulation of immune responses and the promotion of tumor development [11, 12]. The IL-6 receptor (IL-6R) consists of two distinct membrane proteins: the ligand binding strand IL-6Rα (or CD126) that binds to IL-6 and the non-ligand-binding chain glycoprotein 130 (gp130 or IL-6Rβ). There are also two types of IL-6 signaling: classical signaling and trans-signaling [13, 14]. Classical signaling occurs only in some T cells, hepatocytes, mast cells, neutrophils, and monocytes and involves IL-6 binding to IL-6R on the cell membrane to exert anti-inflammatory effects. IL-6 trans-signaling can occur in any cell with membrane-bound gp130 and involves IL-6 binding to sIL-6R to activate signaling through membrane-bound gp130. The classical signaling pathways that bind to receptors through the membrane are primarily regenerative and protective; however, in contrast to the classical pathway, the trans-signaling pathway of sIL-6R promotes inflammation [13]. In the intracellular signaling phase of the trans-signaling pathway [13], a family of tyrosine kinases known as Janus kinases (JAK) is activated after IL-6 binds to the receptor complex. JAK phosphorylates the tyrosine residues in the cytoplasmic region of gp130, which recruits STAT transcription factors that subsequently activate a series of signals that coordinate MAPK and PI3K activation, thereby activating PI3K/Akt/NF-κB for anti-apoptotic and pro-proliferation effects [13, 14].

HIC1 is a transcriptional suppressor that is widely regarded as a tumor suppressor gene. There are 3 widely distributed CpG islands in the promoter region of HIC1 [15]. A number of studies suggest that low expression of HIC1 in cancer tissues may be associated with hypermethylation of the promoter region of the gene, such cancers include breast cancer [16], colon cancer [17], cervical cancer [18], and diffuse large cell type B cell lymphoma [19]. The target genes regulated by HIC1 include fibroblast growth factor binding protein 1 (*FGFBP1*), atonal homolog 1 (*ATOH1*), *CXCR7*, cyclin D1 (*CCND1*) and cyclin-dependent kinase inhibitor 1C (*CDKN1C*) [20] and *p21* [21], which are related to the occurrence and development of various tumors. In our group, HIC1 has been found to inhibit the growth and metastasis of prostate, breast and lung cancer by regulating genes such as *CXCR7* [15], *LCN2* [22], *SLUG* [23] and *IL-6* [24]. Therefore, HIC1 has an important tumor suppressor effect.

There are few reports on the upstream regulation of HIC1. A group of researchers has proposed that p53 is the upstream protein regulating HIC1 expression [20], and another regulator of HIC1 is E2F1 [20]. In addition, another research team has proposed that the expression of HIC1 is also regulated by the level of histone methylation in H3K27 [25].

In this study, we aimed to determine the role of the IL-6/pSTAT3/HIC1 axis in the BrCA environment.

## Methods

### Tissue microarray construction and CAF assessment by immunohistochemistry (IHC)

IHC was performed by using human breast cancer microarrays of formalin-fixed paraffin-embedded (FFPE) tissues (Alianna, Xi an, China), and isolated fibroblasts were stained with antibodies against human α-smooth muscle actin (α-SMA) (ab5694; Abcam, Cambridge, UK) and FAP (ab28244; Abcam). Antibodies (1:100 dilutions) were incubated at 4 °C overnight. Antibody staining was developed using the Vectastain ABC kit (#PK-4000) and DAB (#SK-4100) detection system (Vector Laboratories, CA) and accompanied by hematoxylin counterstaining. Scoring for each immunohistochemistry marker was performed by two experienced technologists who were blinded to the results of other markers or case identity.

### Isolation of primary fibroblasts

CAFs were isolated from human invasive mammary ductal carcinoma tissues, and paracancer fibroblasts (PCFs) were from a region at least 3 cm away from the outer tumor margin in the same patient as the CAFs. Fibroblasts from fibroadenoma (FADs) and non-cancer-associated fibroblasts (NAFs) were isolated from a reduction mammoplasty, in which only normal mammary tissue was detectable. All tissues were minced with scalpels and then enzymatically dissociated in mammary epithelial basal medium (Lonza, USA) supplemented with 2% bovine serum albumin (Promega, USA), 10 ng/mL cholera toxin (Sigma-Aldrich is now Merck KGaA, Darmstadt, Germany), 300 units/mL collagenase (Invitrogen, Carlsbad, CA, USA), and 100 units/mL hyaluronidase (Sigma-Aldrich is now Merck KGaA, Darmstadt, Germany) at 37 °C for 18 h. On the second day, the trypsinized suspension was centrifuged at 700 rpm for 5 min to separate the epithelial and fibroblast cells. The supernatant was collected for centrifugation at 800 rpm for 10 min to pellet the fibroblasts, followed by two washes with DMEM/F12 medium. The cell pellet was resuspended in DMEM/F12 medium supplemented with 5% FBS (GIBCO, Carlsbad, CA, USA) and 5 μg/mL insulin (Tocris Bioscience), plated in cell culture flasks and maintained undisturbed for 2 to 5 days. All tissues were obtained from the Ruijin Hospital with approval of the hospital ethical committee and by the patients’ written informed consent (Shanghai, China).

### Collection of conditioned media (CM) and chemiarray

The CM of all types of fibroblasts was obtained after 48 h of conducting parallel cell culture experiments. The CM samples were then centrifuged at 4000 rpm for 10 min to remove the insoluble substances. Two milliliters of CM were then used for the chemiarray protocol, which is described in the Human Cytokine Antibody Array Kit (RayBiotech, Norcross, GA, USA).

### Enzyme-linked immunosorbent assay (ELISA)

Quantification of IL-6 levels in the supernatants of fibroblasts or breast cancer cells was carried out by ELISA according to the protocol of the human IL-6 Sandwich immunoassay kit (capture IL-6 antibody #MAB206, detection IL-6 antibody #BAF206 and standard rhIL-6 #206-IL; R&D Systems, Minneapolis, MN, USA). All samples were quantified in multiple wells per experiment and repeated three times.

### Cell culture

The human BrCA cell lines MCF7, SK-BR-3, BT-474 and MDA-MB-231 were obtained from the American Type Culture Collection (Manassas, VA, USA) and cultured in Dulbecco’s modified Eagle’s medium (HyClone, Waltham, MA, USA) or RPMI-1640 (HyClone) supplemented with 10% FBS (GIBCO, Carlsbad, CA, USA) and 1% penicillin/streptomycin (GIBCO). Cells were cultured at 37 °C in an incubator with a 5% CO2 atmosphere. Cells were treated with recombinant human IL-6 (#HZ-1019, HumanZyme, Chicago, USA) and STAT3 inhibitor (#S3I-201, Selleckchem, USA) at the indicated concentrations in each manipulation.

### Western blot

Cells were washed 3 times with PBS and treated with RIPA lysis buffer (#89900, Thermo Fisher, Waltham, MA, USA) mixed with protease and phosphatase inhibitor (Roche, Basel, Switzerland). Ten to twenty micrograms of total protein from each sample was resolved on a 10% PAGE gel and transferred to a polyvinylidene difluoride (PVDF, Merck Millipore, Germany) membrane. The blots were then probed with antibodies against GAPDH (1:10000, KangChen, Shanghai, China), STAT3 (1:1000, #4904, Cell Signaling Technology, USA), pSTAT3 (Tyr705) (1:1000, #4903, Cell Signaling Technology, USA), HIC1 (1:5000, #H8539, Sigma-Aldrich, Saint Louis, MO, USA) and cyclin D1 (1:1000, #2978, Cell Signaling Technology), followed by incubation with peroxidase-labeled secondary antibodies. Immunoreactive proteins were detected by enhanced chemiluminescence (ECL) detection kit (Merck Millipore, Germany).

### Cell counting Kit-8 (CCK8) for the cell proliferation assay

Proliferation assays of MCF-7, BT-474, SK-BR-3 and MDA-MB-231 cells treated with different media (supernatant of NAF and CAF) were performed with CCK8 (Dojindo, Rockville, MD). Briefly, cells were cultured in 96-well plastic plate wells in different media for 2 and 4 days, followed by labeling with CCK8 (1:10 dilution) for one additional hour. The absorbance of the samples was measured on a VersaMax Microplate Reader at a wavelength of 450 nm. All experiments were carried out with five parallel wells and repeated 3 times.

### Flow cytometry

BrCA cells were trypsinized and resuspended in PBS containing 2% heat-inactivated FBS and blocked for 10 min with FcR reagent. Then, APC-labeled anti-IL-6Rα antibody (anti-human CD126, #561696, BD Pharmingen, USA) was added and incubated for 30 min on ice in the dark. Thereafter, cells were washed twice with PBS and then analyzed on a FACSCalibur Flow Cytometer (Becton Dickinson, San Jose, USA).

### Cell cycle analysis

Cells in 6-well plates cultured with NAF and CAF were trypsinized, washed and fixed in 70% ethanol for 48 h at 4 °C. The nuclei were stained with propidium iodide (PI, 50 μg/ml) in 1% Triton X-100/PBS containing 100 μg/ml DNase-free RNase, and the DNA content was measured by flow cytometry with the FACSCalibur platform (Becton Dickinson, San Jose, USA). The proportion of cells in the different cell cycle phases was calculated using the ModFit LT program (Verity Software House, USA).

### Colony formation assay

In this assay, one hundred SK-BR-3 cells were plated into each well of a 12-well plate and cultured for 21 days, with an additional equal volume of NAF or CAF supernatant. At the end of the culture period, supernatants were removed and cells were fixed with methanol for 30 min and stained with crystal violet for 30 min. Next, the plates were washed several times with water gently, and images of the optical density of the cells were captured by a digital camera. The stained cell area was measured by Image-Pro Plus 6.0 to determine the cell proliferation level. The MDA-MB-231^shIL-6^ test was performed with a similar method.

### Real-time PCR

Total RNA was extracted from the cells using TRIzol reagent (#15596–026, Invitrogen) and reverse transcribed using the PrimeScript 1st Strand cDNA synthesis kit (#6110A, TaKaRa, China). Real-time PCR was conducted by using the FastStart Universal SYBR Green Master (Rox) (#04913850001, Roche) and Applied Biosystems 7500 Fast Real-Time PCR System (ABI, USA). All results were normalized to the GAPDH internal control. The sequences of the primers that we used were as follows: GAPDH-F: GGAGCGAGATCCCTCCAAAAT, GAPDH-R: GGCTGTTGTCATACTTCTCATGG, IL-6-F: ACTCACCTCTTCAGAACGAATTG, IL-6-R: CCATCTTTGGAAGGTTCAGGTTG.

### IL-6 knockdown and lentivirus packaging

IL-6 knockdown was achieved by constitutively expressing shRNA targeting IL-6 in MDA-MB-231 cells using lentivirus. pLVX-shRNA2 lentiviral vectors expressing the fluorescent protein ZsGreen1 were used (Clontech, Mountain View, CA, USA), and the shRNA sequences were as follows: si-IL-6-1, 5′-CTCAAATAAATGGCTAACTTA-3′. Lentivirus packaging and cell sorting of transfected cells were routinely followed as previously described [24].

## Results

### Upregulation of FAP and α-SMA in BrCA stromal fibroblasts

**Fig. 1:**
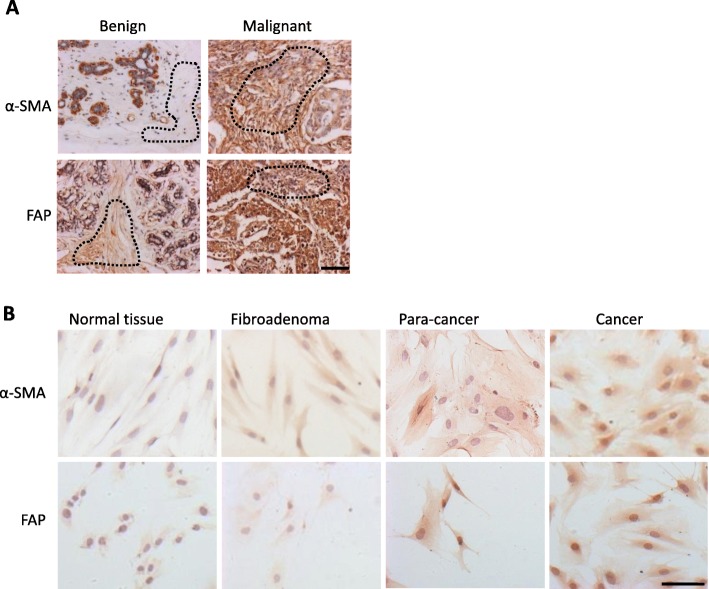
α-SMA and FAP expression in benign or malignant human breast tissues and isolated fibroblasts. **a** Immunohistochemical staining of human breast tissue arrays. Dotted lines indicate the stromal regions. Typical positive samples of malignant stromal tissues were selected and showed higher intensity staining (brown) with α-SMA and FAP antibodies than that of benign tissues. Scale bar, 100 μm. **b** Immunocytochemical staining of α-SMA and FAP in primary fibroblasts isolated from different patients with benign or malignant breast diseases. Scale bar, 100 μm

It is well known that αSMA and FAP are CAF markers in solid tumors [26, 27]. In our work, the two markers were detectable in both fibroblasts of benign and malignant breast tissues (*N* = 96) but still showed a statistically significant difference between benign and malignant tumors in terms of staining intensity (*p* < 0.01). The staining images (Fig. 1 a) demonstrated that fibroblasts in malignant cancer tissues were strongly activated compared with those in benign tissues. The respective median percentages of αSMA- and FAP-positive stroma cells were, respectively, 5 and 20% in benign tissues and 69 and 60% in malignant tissues (*p* < 0.0001 and *p* = 0.002). Therefore, FAP and αSMA were significantly more abundant in the malignant group (Table 1). However, no significant difference was observed among CAFs in different molecular types of breast cancer tissues (*p* = 0.469 and 0.864).

**Table 1:** FAP and αSMA immunohistochemical staining in BrCA Tissue Microarray

*N*=96	Benign (*n*=20)		Malignant (n=76)			
		*p*-value	L-AB (*n*=18)	Her2 (*n*=35)	B-L (*n*=23)	*p*-value
aSMA(-~±)	19 (95%)		7 (9%)	12 (16%)	5 (6%)	
aSMA(+~++)	1 (5%)	<0.0001	11 (15%)	23 (30%)	18 (24%)	0.469
FAP(-)	16 (80%)		7 (9%)	13 (17%)	11 (14%)	
FAP(+)	4 (20%)	0.002	11 (15%)	22 (29%)	12 (16%)	0.864

Furthermore, the isolated fibroblasts were immunostained with anti-FAP and anti-αSMA antibodies. The CAFs expressed higher αSMA and FAP than other benign fibroblasts, including fibroblasts from normal tissue, fibroadenoma and paracancer tissues (Fig. 1 b). These data indicate that the fibroblasts isolated from BrCA tissues were activated and thus utilized for subsequent studies.

### CAFs secrete high levels of IL-6

**Fig. 2:**
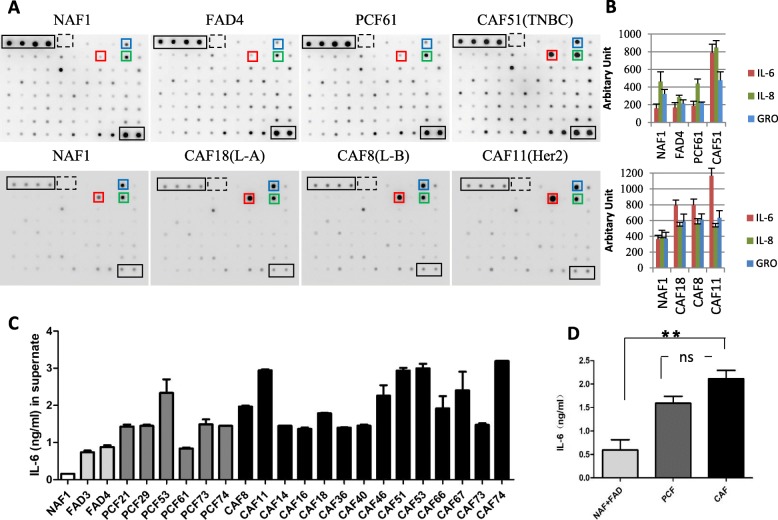
IL-6 is highly expressed in CAFs of breast cancer. **a** Human Cytokine Antibody Array Kits (RayBiotech) were applied to measure the content of 80 cytokines in the CM from diverse fibroblasts. The experiment was performed twice, and the results are shown in two rows. The CM of NAFs served as a control in each experiment. Cytokines upregulated in CAFs and indicated by colored boxes are IL-6 (red), IL-8 (green) and GRO (blue). GRO detects CXCL1, CXCL2 and CXCL3. The enclosed black frames indicate the positive controls, whereas the dashed boxes indicate the negative controls in each membrane. Abbreviations: NAF, non-cancer-associated fibroblast; FAD, fibroadenoma; PCF, paracancer fibroblast; CAF, cancer-associated fibroblast. L-A, luminal A; L-B, luminal B; Her2, Her2 positive; TNBC, triple-negative BrCA. **b** The dot intensity of the different cytokines was quantified by densitometry using ImageJ software (NIH, Bethesda, MD, USA) and normalized to the results. Columns: mean of triplicate experiments; bars: s.d. **c** ELISA results of secreted IL-6 from isolated fibroblasts from accessory breast (white), fibroadenoma (light gray), paracarcinoma (dark gray) and breast cancer (black). The experiments were performed at least three times independently with similar results. Columns, mean of triplicate experiments; bars, s.d. **d** The average concentration of IL-6 was quantified in three groups: accessory breast and fibroadenoma (light gray), paracarcinoma (dark gray) and breast cancer (black). Columns, mean of grouped IL-6 concentration; bars, s.d.

We next collected the supernatant from NAFs, FADs, PCFs and CAFs from four types of BrCA tissues, L-A, L-B, Her2-oe and TNBC, and proceeded with protein microarray analysis to detect the soluble factors secreted by CAFs and other fibroblasts isolated from benign tissues (Fig. 2 a-b). By comparing the soluble factors among all of the fibroblast conditioned media, IL-6 was most significantly upregulated in CAF-conditioned media in contrast to NAF-, FAD- and PCF-conditioned media (Fig. 2 a-b). In addition, GRO (CXCL1, 2, 3) and IL-8 were also markedly upregulated in CAF-conditioned media compared with other conditioned media.

To confirm the protein array results, the supernatant of the fibroblasts isolated from human tissues (numbers were assigned by the collection order) was further examined by ELISA targeting IL-6 (Fig. 2 c). The IL-6 levels in NAF and FAD were lower than those in PCFs and CAFs (Fig. 2 d, *p* < 0.001). We compared the IL-6 level in paired PCFs and CAFs; however, only two pairs of PCFs and CAFs were well isolated in our cohorts, and the IL-6 level was lower in PCF74 than in CAF74, whereas it was comparable in PCF53 and CAF53. In summary, we found that the CM of the CAFs contained slightly more, but not significantly more, IL-6 than that of the PCFs on average (*p* = 0.0687), implying that PCFs might retain CAF-like characteristics.

### CAFs promote breast cancer cell proliferation

**Fig. 3:**
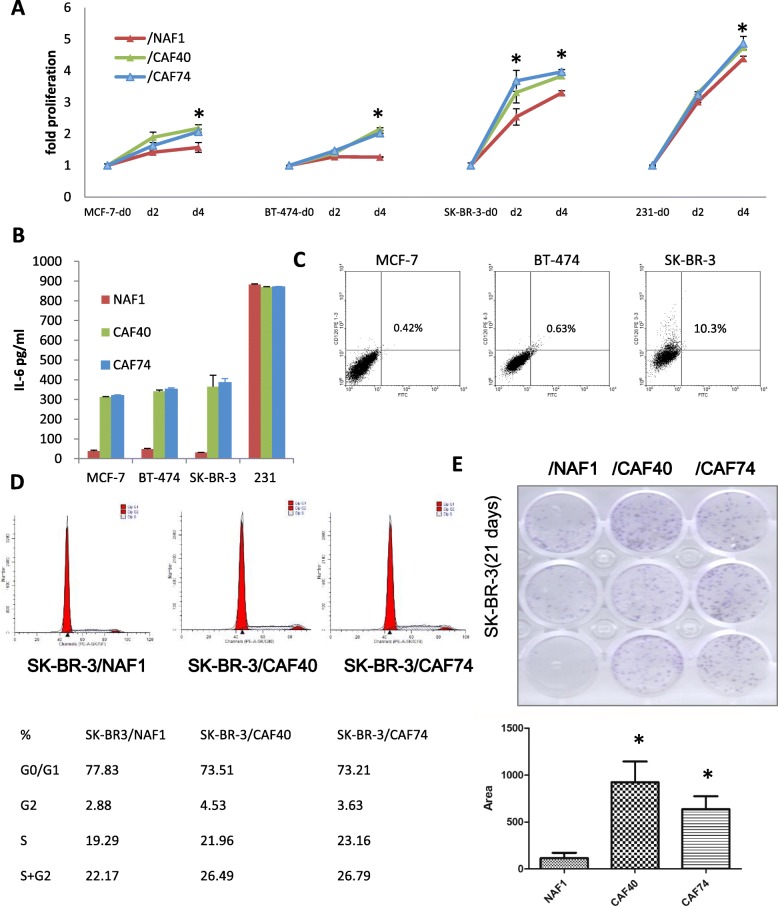
CAFs promote breast cancer cell proliferation. **a** Cell viability assay in MCF-7, BT-474, SK-BR-3 and MDA-MB-231 cells treated with NAF and CAFs CM for 2 or 4 days. Three independent experiments were performed in triplicate. Data are presented as the mean ± s.d. **b** ELISA results of IL-6 from breast cancer supernatant cocultured with NAF1 (red), CAF40 (green) or CAF74 (blue). The experiments were performed at least three times independently with similar results. Columns, mean of triplicate experiments; bars, s.d. **c** Quantification of CD126^+^ breast cancer cells by flow cytometry. **d** Examination of the cell cycle by flow cytometry analysis in SK-BR-3 cells. **e** Colony formation was increased in SK-BR-3 cocultured with CAF40- and CAF74-CM compared to NAF1-CM. The dotted area of the stained cell colonies was quantified by Image-Pro Plus software (Media Cybernetics, USA) and normalized to the results. Columns, mean of triplicate experiments; bars, s.d.

To explore the function of CAFs in BrCA, four different types of BrCA cell lines, MCF-7, BT-474, SK-BR-3 and MDA-MB-231, were cocultured with CM from NAF1, CAF40 and CAF74. NAF1 was isolated from an operable patient with accessory breast. CAF40- and CAF74-CM significantly induced higher proliferation of all BrCA cell types compared with NAF-CM (Fig. 3 a), but most remarkably in SK-BR-3 cells. The cocultured CM from cancer cells and fibroblasts were coordinately collected. Expectedly, the IL-6 concentration remained significantly elevated when MCF-7, BT-474 and SK-BR-3 cells but not MDA-MB-231 cells were cocultured with CAF40- and CAF74-CM compared with NAF1-CM (Fig. 3 b) due to abundant IL-6 in MDA-MB-231 cells (Fig. 5 a). We then detected the expression of IL-6 receptor alpha (CD126) in three low IL-6-secreting BrCA cell lines and found that SK-BR-3 expressed the highest amount of CD126 (10.3% positive) in the cell membrane (Fig. 3 c). Therefore, SK-BR-3 cells were used in the following experiments.

SK-BR-3 cells were cocultured with NAF1, CAF40 and CAF74 for 24 h, and the proportion of S and G2 phase cells was augmented when SK-BR-3 cells were cocultured with CAFs compared with NAF1 cells (Fig. 3 d). In addition, the supernatant of the CAFs also promoted SK-BR-3 colony formation compared with the NAF1 supernatant (Fig. 3 e).

### CAF-derived IL-6 decreases HIC1 expression in SK-BR-3 cells

**Fig. 4:**
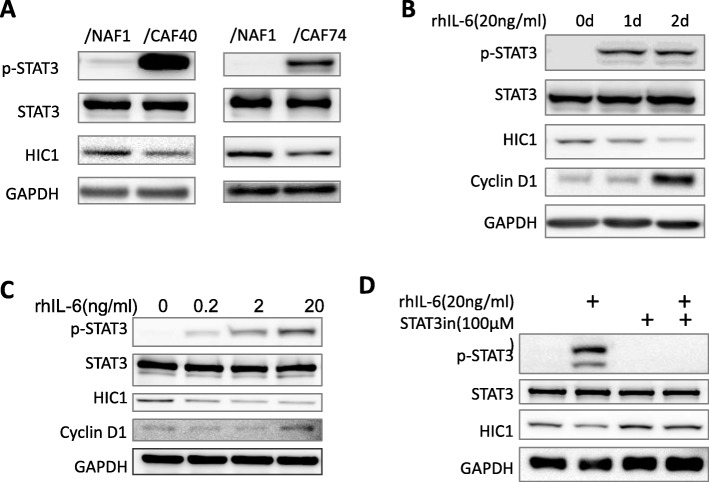
CAF-derived IL-6 decreased HIC1 expression in SK-BR-3 cells**.** HIC1 was decreased in SK-BR-3 cells cocultured with CAF40 and CAF74 compared to cells cocultured with NAF1. **b-c** HIC1 was decreased in SK-BR-3 by rhIL-6 in a time- and dose-dependent manner. **d** The decreased HIC1 induced by rhIL-6 was completely restored by STAT3 inhibition

When cocultured with CAF40 and CAF74, SK-BR-3 displayed activation of STAT3 and decreased levels of HIC1 compared to cells cocultured with NAF1 (Fig. 4 a). We then used rhIL-6 to mimic the CAF-derived IL-6 in order to avoid interference from other factors. rhIL-6 indeed decreased HIC1 expression in a time- and dose-dependent manner and was accompanied by increased cyclin D1 (Fig. 4 b-c). Moreover, the decreased HIC1 induced by rhIL-6 was completely restored by STAT3 inhibition (Fig. 4 d). We next focused on the IL-6/STAT3/HIC1 axis in BrCA cell lines.

### IL-6/pSTAT3/HIC1 axis in MDA-MB-231 cells

**Fig. 5:**
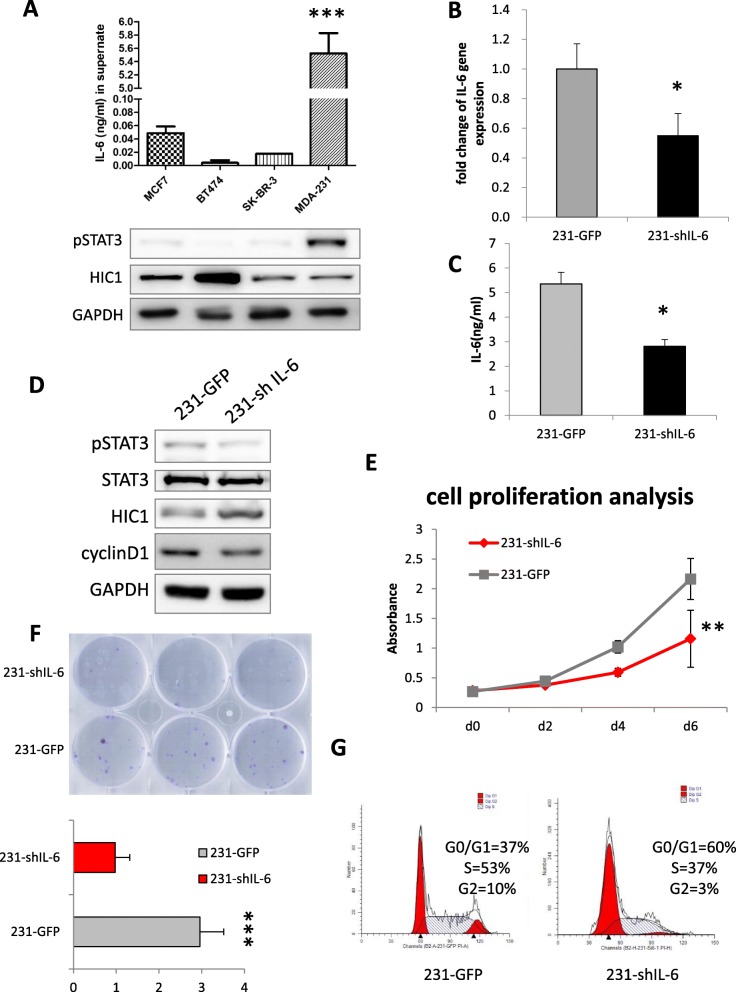
The IL-6/pSTAT3/HIC1 axis in MDA-MB-231 cells. ELISA results of secreted IL-6 and expression of pSTAT3 and HIC1 in the BrCA cell lines. **b** Real-time PCR was used to examine the IL-6 knockdown effect in MDA-MB-231 cells. **c** ELISA results to confirm the decreased expression of IL-6 in 231-shIL-6 cells. **d** HIC1 expression was increased in sh-IL-6 cells. **e** Cell viability assay in 231-shIL-6 cells for 2, 4 and 6 days. **f** Colony formation was decreased in 231-shIL-6 cells after 10 days. **g** Examination of the cell cycle by flow cytometry analysis in 231-shIL-6 cells

To verify the IL-6/pSTAT3/HIC1 axis in BrCA, we examined IL-6 expression in various types of BrCA cells and found that IL-6 was highly secreted by MDA-MB-231 cells (Fig. 5 a). Furthermore, pSTAT3 was highly activated and HIC1 was weakly expressed. Subsequently, we knocked down IL-6 gene expression by lentiviral vectors (Fig. 5 b-c). HIC1 was found to be increased in sh-IL-6 cells (Fig. 5 d). The proliferation ability of 231-shIL-6 cells was dramatically lower than that of the control group (Fig. 5 e), and the cell colony formation was clearly inhibited (Fig. 5 f). Cell cycle detection showed that the proportions of 231-shIL-6 cells in the S and G2 phases decreased significantly (37% vs 53 and 3% vs 10%, respectively) (Fig. 5 g).

## Discussion

Increasing evidence suggests that the conversion of stromal fibroblasts into CAFs plays a significant role in BrCA development [9, 28]. In our study, it was found that stromal fibroblasts isolated from four molecular subtypes of BrCA tissues secreted high levels of IL-6 compared to noncancer patient tissues. It has already been demonstrated that CAFs secrete abundant IL-6 in BrCA [12]. Here, we further examined the CAFs from four subtypes of BrCA and demonstrated that the CAFs express high levels of IL-6 in all types of BrCA.

We also found that fibroblasts isolated from the peripheral tissue of the cancers showed comparable levels of IL-6. This finding is consistent with a previous report that fibroblasts present in histologically normal surgical margins (interface zone fibroblasts, INFs) of BrCA patients exhibited a tumor-promoting phenotype [7]. Although neither PCFs nor INFs were considered as cancer-associated fibroblasts, the PCFs were treated as normal or control group in some studies [7, 29], we think that the bona fide role in BrCA requires further investigation.

In addition to IL-6, IL-8 and GRO (including CXCL1, 2 and 3) were also found to be higher in CAFs than in other benign fibroblasts. It is known that either IL-8 or GRO function as growth factors or chemokines. When we stimulated the breast cancer cell line SK-BR-3 with rhIL-8 and rhCXCL1, HIC1 expression was not decreased (data not shown). Thus, IL-8 and GRO were not analyzed in this study. Nevertheless, we could still not exclude both roles in BrCA development.

By stimulation with rhIL-6, we found that MCF-7 and BT-474 showed decreased expression of HIC1 at both the protein and mRNA levels (data not shown), and SK-BR-3 exhibited decreased HIC1 protein but increased mRNA. Herein, we examined the promoter methylation level of the HIC1 gene and protein ubiquitination level after rhIL-6 stimulation, and no obvious changes were found (data not shown). Therefore, we speculate that there are different IL-6-mediated HIC1 regulatory mechanisms in different breast cancer cell lines.

In our previous study, HIC1 was found to be a suppressor of IL-6 in non-small cell lung cancer [24], and HIC1 was found to be weakly expressed in the TNBC cell line MDA-MB-231 [22]. In this paper, we found that IL-6 could inhibit HIC1 expression. Therefore, IL-6 and HIC1 should be reciprocally regulated by each other. Their regulation mode and role in cancer deserve to be investigated in future work.

Based on our findings, we discovered that all types of CAFs from BrCA tissues secrete high levels of IL-6 that promote BrCA development and that the IL-6/pSTAT3/HIC1 axis plays an important role in BrCA development. Additionally, in BrCA cells enriched with IL-6, IL-6 is able to decrease HIC1 expression by autocrine signaling, which causes a more aggressive phenotype and poor prognosis.

## Conclusions

**Fig. 6:**
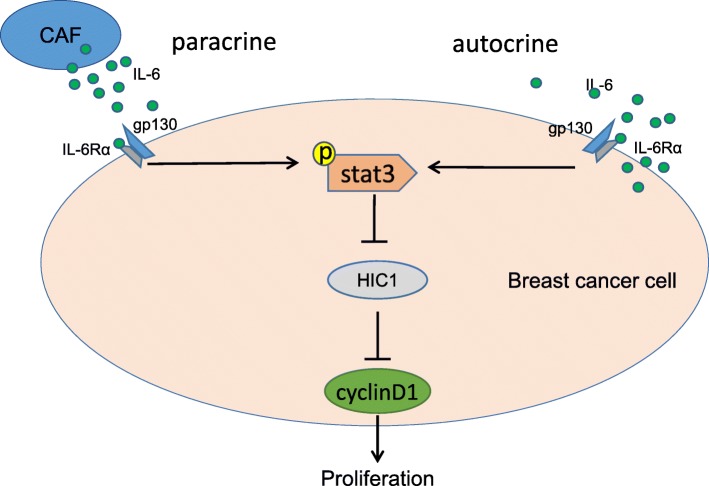
Schematic of the potential mechanisms by which IL-6 activates STAT3 and downregulates HIC1 expression through paracrine or autocrine signaling

Increased IL-6 was found in the supernatant of isolated CAFs, which suppressed HIC1 expression in cancer cells and promoted BrCA cell proliferation. By stimulation with rhIL-6, STAT3 was phosphorylated, and HIC1 was decreased in the BrCA cells, while STAT3 inhibition completely rescued HIC1 expression (Fig. 6). Moreover, in high-IL-6-secreting MDA-MB-231 breast cancer cells, HIC1 was restored upon knocking down IL-6 expression, accompanied by decreased STAT3 activity. These findings indicate that IL-6 downregulates the tumor suppressor HIC1 and promotes BrCA development in the tumor microenvironment through paracrine or autocrine signaling.

## Data Availability

The datasets used and analyzed during the current study are available from the corresponding author on reasonable request.

## Abbreviations

BrCA: Breast cancer
CAFs: Cancer-associated fibroblasts
FADs: Fibroblasts from fibroadenoma
GRO: Includes CXCL1, CXCL2 and CXCL3
Her2-oe: Her2-overexpressing
HIC1: Tumor suppressor hypermethylated in cancer 1
IL-6: Interleukin-6
L-A: Luminal A
L-B: Luminal B
NAFs: Non-cancer-associated fibroblasts
PCFs: Paracancer fibroblasts
rhIL-6: Recombinant human IL-6
STAT3: Signal transducers and activators of transcription 3
TNBC: Triple-negative breast cancer
